# Human Papillomavirus Infection and Vaccine Uptake Among Males in the United Arab Emirates and the Wider Middle East and North Africa Region: A Narrative Review

**DOI:** 10.7759/cureus.96195

**Published:** 2025-11-06

**Authors:** Humaid AlKaabi, Raya Abu-Khalaf, Sandra Abu-Khalaf, Layan Abu-Khalaf, Yahia Khalil

**Affiliations:** 1 Internal Medicine, Zayed Military Hospital, Abu Dhabi, ARE; 2 Medicine, Royal College of Surgeons in Ireland, Dublin, IRL; 3 Internal Medicine, Jordan Hospital, Amman, JOR; 4 Geriatric Medicine, Blackpool Victoria Hospital, Blackpool, GBR; 5 Internal Medicine, St Mary's Hospital, Isle of Wight NHS Trust, Newport, GBR

**Keywords:** health awareness, hpv infection, hpv vaccine, male population, middle east, north africa, united arab emirates, vaccination coverage

## Abstract

The human papillomavirus (HPV) is one of the most common sexually transmitted diseases (STIs) worldwide. It is responsible for a significant malignancy burden in both men and women. This is not only limited to its association with cervical cancer in women, but it is an increasingly recognized causative agent in oropharyngeal, anal, and penile carcinomas in men. Despite this, vaccination efforts in the United Arab Emirates (UAE) and the Middle East and North Africa (MENA) have almost solely targeted women, while there is a wide gap in the protection of males. This review discusses the available evidence regarding HPV infection and vaccination among males in the UAE and the wider MENA region. Despite heightened awareness among some populations, such as university students and health trainees, HPV vaccine uptake remains low. Cultural and religious considerations, parental hesitancy, concerns about adverse effects, and widespread misinformation, often driven by social media, are also barriers to vaccination. Physicians are also uncertain about the role of the vaccine in boys, and a majority of national programs have not yet recommended boys. Systemic obstacles, including cost and unequal availability, also restrict access. In the meantime, progress is in sight: the UAE and a few adjacent countries are beginning to roll out HPV vaccination more broadly, and increased interest in rebranding the vaccine as a cancer prevention, as opposed to a sexual health issue, is growing. Closing the current gaps in awareness, access, and policy will be necessary to mitigate the growing burden of HPV disease among women and men across the MENA region.

## Introduction and background

Human papillomavirus (HPV) is one of the most prevalent STIs in the world [1]. There are more than 200 types of HPV, including those that cause cancer. Cervical cancer is mainly caused by HPV, with HPV types 16 and 18 accounting for over 70% of cervical malignancies and precancerous cervical lesions [1]. In addition to cervical cancer, HPV is linked to malignancies of the vulva, anus, vagina, penis, and oropharynx [1]. In males, HPV-related malignancies are rising, including oropharyngeal, anal, and penile cancers, particularly in immunosuppressed and at-risk populations [2]. This increase is primarily attributed to HPV, specifically HPV-16, and is happening despite a decrease in smoking-related throat cancers. Additionally, globalization's rapid changes in lifestyle have affected sexual behaviour, particularly in younger generations, leading to increased transmission of HPV infection and other sexually transmitted illnesses [2]. 

The purpose of HPV vaccinations is to produce acquired immunity against infection by specific HPV types [2]. The HPV vaccine was initially made available in 2006 and was initially meant for females developing cervical cancer, but has, over time, been expanded to male populations [2]. This study focuses on the current status of HPV and the HPV vaccine among the male population in the United Arab Emirates (UAE) and the wider Middle East and North Africa (MENA) Region. This analysis investigates statistical data patterns coupled with community-level obstacles while also exploring policy interventions and justifications behind addressing gender-based prejudices in HPV protection measures. HPV vaccination for males remains an underutilized global strategy largely due to social resistance experienced in regions with traditional gender norms, including the MENA. This study also advocates for making gender-neutral HPV vaccination part of standard adolescent immunization programs throughout the MENA region to deliver fair disease protection for all. 

The HPV vaccine has shown promise in lowering the prevalence of cervical cancer by specifically targeting high-risk HPV types 16 and 18. Of the World Health Organization (WHO) member states, 47 (24%) had included the HPV vaccination in their national immunization program for boys as of 2022 [3]. In males, the vaccines have been demonstrated to lessen their chance of developing HPV-induced precancerous lesions and progression to anal and penile cancer [3]. Currently, the HPV vaccine formulations licensed include three bivalent (protecting against two varieties of HPV), two quadrivalent (protecting against four), and one nonavalent (protecting against nine) vaccine [1]. All are very effective or have fulfilled immunobridging requirements, and they all have outstanding safety profiles [1]. Gardasil has also demonstrated efficacy in preventing high-risk male HPV types 16 and 18. For males, the Gardasil and Gardasil 9 vaccinations have been licensed; however, Cervarix, a third HPV vaccine, varies by jurisdiction. Cervarix does not offer genital wart protection, in contrast to the Gardasil-based vaccines [1]. For men who have sex with men, who are more likely to develop anal cancer, penile cancer, and genital warts, Gardasil is also utilized [1]. They also reduce the risk of HPV-related penile lesions [4]. Additionally, the quadrivalent and nonavalent vaccines offer nearly total protection against genital warts, commonly referred to as anogenital warts [4]. This review aims to discuss the current evidence regarding HPV infection and vaccination uptake among males in the UAE and the wider MENA region. This is reviewed by highlighting key barriers, including sociocultural and systemic barriers, and expanding evidence on the ongoing efforts and public health strategies to improve HPV vaccine coverage. 

This narrative review was conducted using a search in PubMed, Semantic Scholar, and Scopus to identify peer-reviewed studies, review articles, and regional reports published between 2006 and 2025. Keywords used included: "HPV,” “vaccination,” “males,” “UAE,” and “Middle East and North Africa.” We selected English language publications that were relevant to HPV infection and vaccination in the UAE and MENA region. No formal inclusion or exclusion was applied due to the narrative design of the review.

## Review

Current epidemiology of HPV within males in the MENA and UAE

At present, research indicates cervical cancer remains the most commonly studied type of HPV linked malignancy [1]. The epidemiology of HPV in males is not well established in the wider MENA region due to the lack of surveillance systems and limitations to sexual health discussions [5]. Nevertheless, existing data point to a rising incidence of HPV-related conditions in both men and women. Among the Gulf Cooperation Council (GCC) countries that consist of Bahrain, Kuwait, Oman, Qatar, Saudi Arabia, and the UAE, studies indicate that HPV positivity rates differ significantly [6]. According to the Saudi Med J study [7], cervical cancer is a prevalent cancer in Saudi Arabia, being placed at the 11th rank in the overall prevalence of cancer and the ninth rank among women between the ages of 15 and 44 in 2021.

Another study by Bruni et al. [8] published in the Lancet Global Health reveals that nearly one in three male individuals over the age of 15 has at least one type of genital HPV. Of concern, one in five has one or more of the so-called high-risk, or oncogenic, HPV types. The pooled prevalence of any HPV and high-risk HPV globally was 31% (27-35) and 21% (18-24), respectively [8]. The most common HPV genotype was HPV-16, 5% (95 percent CI 4-7), and HPV-6 was 4% (3-5) [8]. The prevalence of HPV was found to be high among young adults, with a peak in the age group of 25 to 29 years, after which it remained relatively constant or decreased [8]. A systematic review [5] emphasized that HPV is one of the most frequently reported STIs across regions in the MENA at 23%, albeit likely underestimated due to cultural stigma, low screening, and sampling biases. HPV causes nearly all cervical cancers worldwide, while it creates 90% of anal cancers and over 70% of oropharyngeal cancers that develop in men [1]. However, new trends show HPV-related oropharyngeal cancers are surpassing cervical cancer cases in certain high-income countries, a pattern that likely will emerge in MENA during the upcoming years [8]. Hospital-based studies report lower apparent MENA prevalence compared to global averages, but this likely reflects under-detection rather than true lower incidence [8]. A scoping review [9] focusing on GCC men and women found HPV prevalence in UAE males ranged widely from ~14.7% up to 88%, with HPV-16 being the most common high-risk genotype and HPV-11 and HPV-6 common low-risk types. The highest rate is 31.3% in Qatar, followed by Bahrain at 20%, Saudi Arabia at 17.2%, and the UAE at 14.7% [9]. More findings show that HPV prevalence is high in men over the age of 15 years and support that sexually active men, regardless of age, are an important reservoir of HPV genital infection [9]. 

Accurate data for male HPV infections is limited, but evaluations in developed countries indicate that roughly half of sexually active men will develop an HPV infection over their lifespan [10]. Risk factors include a high number of sexual partners and early sexual debut, with men frequently unaware that vaccination applies to them [11]. Data on HPV prevalence rates among MENA men remains uncertain because of social stigma, along with insufficient testing and the absence of standardized screening procedures. According to a narrative review by Kim and Bae [12], the scarcity of information about male HPV infection reinforces misconceptions that males face fewer consequences or serve only as disease transmitters to females but are not affected.

Mapping HPV transmission patterns across the MENA region in the past has been difficult because of regional cultural restrictions against sexual activity before marriage. Epidemiological studies indicate that youth engage in both heterosexual and same-sex sexual activity discreetly despite Islamic teachings, which encourage abstinence before marriage. Gamaoun [13] conducted a thorough review in 2018 that included 18 studies conducted in nine Arab countries. States and subpopulations differed in their knowledge and awareness of HPV infection, ranging from 4.2% to 97.0% [13]. The subgroup of parents of the general population had the lowest percentages (varying from 4.2 to 18.0%), whereas the segment of physicians had the highest rate (97.0%) [13]. Only 8.4% of parents had sufficient understanding and awareness of the link between HPV infection and cervical cancer [13]. Depending on the nation, parents' awareness of the HPV vaccine ranged from 14.2% to 34.2% [13]. Although almost 99% of parents said they approved of the vaccination, there was not enough movement to take action [13].

The combination of premarital sexual behaviour with sparse condom usage and limited knowledge of STIs generates an increasing yet unacknowledged danger for HPV transmission: healthcare providers, along with the general public, show a limited understanding of this matter. Research [13] showed that HPV recognition can be low in regional surveys, while cancer association awareness was as low as 8.4%. Many healthcare professionals maintain incorrect assumptions about how the vaccine benefits men because they continue to think it functions exclusively to protect against cervical cancer [14]. The epidemiological data from the UAE and MENA region demonstrate that an important mismatch exists between HPV-related risks and preparedness measures in public health. These estimates highlight how crucial it is to include men in all-encompassing HPV prevention programs to lower male HPV-related morbidity and death and eventually eradicate cervical cancer and other HPV-related illnesses.

HPV vaccine uptake in the GCC and MENA

Existing data on HPV vaccine uptake among males in the GCC countries reveals a persistent gap between awareness, policy implementation, and actual vaccination coverage. Since 2010, the vaccination has been made available in Saudi Arabia at major medical facilities, either for free or at a cost to women who have a valid prescription [15]. The vaccination, which is usually given to females between the ages of 11 and 26 in three doses over six months, is highly successful in preventing cervical cancer and lowering the prevalence of several disorders linked to HPV [15]. However, the vaccination in males is still low [15]. Historical HPV prevention campaigns have centred on female populations because of cervical cancer risks, yet they have disregarded the substantial health consequences for males. Notably, only the UAE and Libya currently include males in their national immunization programs [5]. Vaccine availability varies across the GCC and MENA. For example, Oman has not yet included the HPV vaccine in its Expanded National Immunization Program, limiting its availability to a few private institutions [6]. The MENA regions display distinctive characteristics that shape their public health landscape. The UAE leads progressive reforms for gender-neutral HPV vaccination, but ongoing sociological and religious obstacles and educational limitations challenge wider implementation across the MENA region. HPV vaccination programs for boys face opposition because some stakeholders view the virus as female-specific and dismiss precoital sexual behaviour. 

A 2024 cross-sectional survey [16] conducted across Bahrain, Kuwait, Oman, Qatar, Saudi Arabia, and the UAE found that male vaccination rates remain relatively low, with the UAE leading at 18.9%, followed by Qatar (5.8%) and Saudi Arabia (4.6%). While vaccine awareness in Saudi Arabia (58.8%) and the UAE (49.6%) appears relatively high, these figures do not translate into proportionate uptake, highlighting the inadequacy of awareness alone in driving vaccination behaviour. Despite the UAE introducing a gender-neutral school-based HPV vaccination program in recent years-after initially targeting females in 2008-coverage among adolescent boys remains under 20%, indicating a disconnect between policy and practical outcomes [16]. A study by Farsi et al. [14] further emphasizes this trend; for instance, a 2018 study among male medical students in Saudi Arabia found that although 74% had heard of HPV, only 42% were aware of the vaccine, and just under half expressed willingness to be vaccinated. Broader MENA region studies [13] reinforce that knowledge and vaccine acceptance increase when individuals are informed; however, uptake is consistently impeded by structural factors such as cost, lack of national mandates, weak provider recommendations, and sociocultural resistance. 

Barriers to HPV vaccine uptake among males in the UAE and MENA region

The MENA region demonstrates slow developments in terms of male acceptance rates of gender-neutral HPV vaccination, even after recent worldwide recognition of the usefulness of this approach [15]. The history of HPV vaccination inthe UAE started in 2008 when the vaccination was introduced in girls, and in 2023, girls were extended to boys aged 13 to 14 as part of the gender-neutral approach [15]. A mix of sociocultural barriers, religious barriers, educational barriers, and systemic barriers affects uptake in a region with conservative sexual health practices [5]. 

Cultural and Religious Taboos

Religious objections combined with concerns about side effects from vaccine usage emerged as primary avoidance reasons in Islamic countries. Darraj et al. [17] conducted a study on knowledge and acceptance of the HPV vaccine and demonstrated that religious objections accounted for 30% of opposition to the vaccine. Interestingly, the review demonstrated that religious beliefs did not necessarily influence overall vaccine uptake, but they did have implications for vaccine acceptability. The research located various misconceptions and beliefs regarding HPV vaccination. Some of such misconceptions were the vaccine being a source of ethnic cleansing, promotion of sexual immorality, desecration of religious practices, or an ulterior way of introducing haram components into the bodies of Muslims. Similar findings were reported in a review by Hendaus et al. [18] in UAE and Qatar studies, which showed that women who believed the HPV vaccine was unnecessary for their children often based their decisions on religious and cultural beliefs. Societies that follow Islamic beliefs that focus on chastity and modesty perceived the HPV vaccine to be linked with discussions of sexual activity, which may be culturally sensitive. Although Islam supports preventive measures, including vaccinations, some believers perceived the marketing of HPV vaccines as a subtle endorsement of unacceptable sexual behaviors [19].

Sexuality and STIs are subjects that are not openly explored throughout the MENA region due to religious reasons. Since the common belief is that the religious faithful will not engage in sexual activity before marriage, this deems the HPV vaccine unnecessary. Islam forbids having sex before marriage, and individuals in Islamic nations are expected to adhere to these principles. Concerns about addressing sexual health with minors further exacerbate this impression of the vaccine, which directly adds to the region's low immunization rates [19]. Parents and adolescents decline HPV vaccination because they want to distance themselves from any perception that they support promiscuous behaviour.

Another noteworthy issue concerns the requirement for vaccinations that the items be confirmed to be halal [20]. Islamic jurisprudence in Kuwait, Qatar, Saudi Arabia, UAE, Oman, and Bahrain states that Muslims are prohibited from utilizing any chemicals or medical remedies that come from haram sources, such as pigs or their derivatives. Due to their belief that the vaccine is derived from pig experiments, the majority of Muslims choose not to receive vaccinations [21]. When a vaccine's "halal" certification is in doubt, parents usually turn to homeopathic or alternative treatments [21]. Another study [21] further noted a correlation between a lower vaccination rate and the use of complementary medicine by the older generation, which demonstrates their ambivalence to adopting newer advances in medicine.

Low Health Literacy

A significant theme throughout the literature is unfounded concerns regarding vaccine safety and vaccination efficacy. In Saudi Arabia, the review by Alhusayn et al. [22] found that hesitancy is widespread due to such factors as the fear of adverse effects and a low level of awareness of HPV itself. Similar observations were replicated in Qatar, where Hendaus et al. [18] noted that some participants were concerned that the vaccine was developed mainly to generate profit, indicating a profound mistrust. These problems are compounded by a general lack of understanding and misunderstandings of personal risks. Saqer et al. [23] confirmed that a significant number of male citizens in the UAE refused vaccination, arguing they were either too old or at no risk against the virus. Some knowledge gaps still exist even among well-informed groups, such as university students and healthcare trainees. A Saudi study [14] revealed that while a majority of participants acknowledged HPV as a serious health issue, only 63.7% believed the vaccine to be highly effective, and 13.7% cited unawareness as a vaccination barrier. Alarmingly low knowledge scores were also noted among Saudi university students (mean score 1.99/10), with over 60% believing no vaccine exists and only 39.6% aware that HPV can affect both genders [14].

Parental Influence as a Barrier to HPV Uptake

Parental concerns, ranging from safety to religious beliefs and ignorance, greatly influence the uptake of vaccines in children. In a study in Saudi Arabia [24] where 67% of parents feared serious side effects, and 41% expressed scepticism about the vaccine's effectiveness. A systematic review by Alshahrani et al. [6] on parental perspectives on HPV vaccination in GCC levels of awareness was low to modest, as awareness of HPV and its association with cervical cancer ranged from 11% to 68%. Alshahrani et al. [6] further noted that Saudi Arabia had a negative attitude regarding immunization, whilst the UAE and Qatar had more favourable views. The main obstacles were cultural attitudes, informational gaps, and safety concerns. The choices of parents to vaccinate their children against HPV depend on their attitude towards the vaccine, as well as socioeconomic position and general knowledge of the need [25]. As indicated in a study by Hendaus et al. [18], parental factors like the religion of parents, their level of education, their age, gender, occupation, and the number of children a family had were found to be significant factors that influence the uptake of HPV vaccination in children. Moreover, lower income levels and poverty are linked to low HPV vaccine uptake rates, accompanied by cultural norms [22]. Parental attitudes, understanding, and health behaviours are also influenced by concerns over side effects and the long-term safety of vaccines [26]. Al Saedi et al. [27] studied parental awareness, attitude, and acceptability of the HPV vaccine in Jeddah, Saudi Arabia, and demonstrated that vaccine hesitancy was primarily due to fear of adverse effects (33.1%) and insufficient information (19.7%). Statistical analysis revealed significant correlations between vaccine acceptability and parental education level, employment status, and personal vaccination history [27].

Misinformation 

Misinformation remains a significant barrier to HPV vaccine uptake in the MENA region, particularly among male populations. In a review of the contributors to vaccine hesitancy in Oman, Alsulami [28] concluded that less than 25% of Omani adults had the correct knowledge about HPV and its associated health outcomes. Those who took part in the survey showed a considerable degree of misinformation by having unrealistic perceptions of the possible outcomes of the vaccine. Approximately 19% and 17% of the participants agreed or strongly agreed that the HPV vaccine is being promoted for profit by pharmaceutical companies and that it is unsafe [28]. According to Taumberger et al. [29], digital platforms contribute substantially to widespread misinformation, which represents a significant obstacle to vaccine acceptance. Social media users frequently encounter baseless conspiracy theories along with anecdotal harm reports and misinformation regarding infertility, which generates vaccine skepticism. Some of the prevalent myths regarding HPV that were elicited were that vaccinations induced severe adverse events like autoimmune diseases and death, boys and men do not get cervical cancer, so they do not need a vaccine, and that natural HPV infection already creates a protective antibody response, so there is no need for vaccination [29]. The absence of trustworthy cultural information deepens public anxiety and misinformation. As Alkhalili et al. [30] noted, health literacy across the MENA region demonstrates significant disparities, with sexual health education missing from almost every school curriculum. Young people and their parents depend on inaccurate information from peers, unverified internet sources, and religious leaders when formal education is not available. The lack of structured educational programs gives rise to misconceptions that claim HPV disease attacks only women while ignoring the preventative value of vaccination for religious followers who practice proper sexual behaviours [28].

Healthcare Provider Knowledge Gaps and Systemic Barriers

Healthcare providers maintain essential positions in vaccine promotion, yet numerous healthcare professionals demonstrate limited awareness regarding HPV vaccine advantages for males. A study conducted in Bahrain [31] discovered that numerous doctors refrain from recommending the vaccine to male patients because they receive minimal education, operate under unclear national protocols, and are worried about patient reactions. Farsi et al. [14] studied the understanding of HPV male vaccination among students preparing to join the workforce. The study found that despite rising cases of HPV-related oropharyngeal cancers, male medical students had low HPV knowledge. About 74% had heard of HPV, but only 42% were aware of the vaccine, and only roughly 49% were willing to receive it [14]. Healthcare professionals often fail to take advantage of educational opportunities when patients present for health check-ups due to various reasons, including avoiding the risk of upsetting patients and appearing judgmental. This creates a need for the promotion of HPV vaccines by empowering care providers and making them agents for promotion to achieve adequate disease prevention.

Limited vaccine accessibility due to systemic challenges also affects the capacity of healthcare providers to routinely administer it to potential candidates. In the MENA region, HPV vaccination programs for males are not widely implemented, though some countries are beginning to introduce them. Despite the UAE government's recent decision to include 13-14-year-old boys in its vaccination program, there are various distribution obstacles. While the 9-valent HPV vaccine (Gardasil 9) and other HPV vaccines are approved in the region, national immunization programs often do not include boys, and even when they do, coverage is often low [32]. National immunization programs across certain countries exclude HPV vaccines for boys, which forces families to buy them privately while dealing with financial barriers, social stigmas, and limited availability. 

Efforts and advances in male HPV vaccination in the UAE and MENA region

While the vaccination trends are far below the ideal, there are signs that MENA is catching up on HPV prevention through vaccination. The landscape of HPV vaccination in MENA has seen significant changes in recent years, particularly in more affluent nations such as the UAE, Qatar, and Saudi Arabia. These countries have integrated HPV vaccines into their national immunization programs, making them available for children as young as 12, targeting both boys and girls. Their vaccines are typically provided within two doses at 0 and 6 to 12 months [15]. There is also the emergence of new technologies in the development of vaccines, with the potential for more effective and lasting vaccines. Some are even exploring vaccines that could treat HPV infections, not just prevent them [33]. 

Social sensitization is on the rise, where communities are trying to break cultural and religious stereotypes that have been used to prevent open dialogue on sexual health concerns. Education is being employed to address cultural beliefs, misconceptions, and logistical challenges, especially in remote areas, that pose obstacles. Efforts are also made to reach the rural areas, which often have lower coverage compared to urban regions, indicating an uneven distribution of the vaccine. The success of HPV vaccination campaigns relies on reframing the vaccine as an anti-cancer protection approach rather than a sexual disease prevention in the MENA region conservative contexts [34]. Through sensitive execution and scientific precision, gender-neutrality policies achieve health-based goals while protecting social equity. Efforts have been made to stop viewing religion as a barrier, but a potential facilitator to HPV prevention. Adherence to religious principles, such as abstaining from premarital sex or believing that religiously based circumcision reduces HPV prevalence, plays a role in shaping one's health-related choices. Some models have started framing vaccines as aligning with the religious requirement to preserve human life and dignity and to protect women by avoiding causing them preventable harm [34].

Nevertheless, the MENA region is not uniform, with countries in the region not equally achieving implementation success due to financial, cultural, or even educational impediments. However, governments continue to be more proactive, with quite a number of them relying on the advice of international health bodies that seek to assist them in making their HPV vaccination programs more responsive. The efforts are focused on shaping their HPV vaccination policies, focusing on educating healthcare providers to improve vaccine uptake. This growing recognition marks a significant step toward setting standards for gender-neutral HPV vaccination, thereby increasing overall coverage rates in the MENA region.

Recommendations

*Adopting Gender-Neutral Vaccination Programs * 

A public health strategy that includes comprehensive gender-neutral vaccination represents both an ethical requirement and an effective fundamental pillar for disease prevention. According to evidence research and guidelines from the WHO [3], both boys and girls aged 9 to 14 should receive HPV vaccinations. Multiple simulations indicate that herd immunity needs at least 80% population participation to succeed, yet this benchmark becomes unreachable if boys are excluded from vaccination programs. The program's overall benefits become compromised because heterosexual males remain capable of virus transmission after selective female vaccination procedures. Vaccinating male and female populations equally will reduce healthcare system strain through decreased rates of HPV associated diseases while trimming future treatment costs. Gender-neutral messaging on HPV enables universal healthcare by eliminating the bias inherent to sexual activity and targeted population, and establishes it as a standard of care.

Increasing Vaccine Availability, Access, and Distribution

The solution to achieving underserved populations in low-income communities should be through national policies offering free or subsidized vaccines. Also, school-based vaccine delivery systems should be made possible. Healthcare providers working at schools can increase vaccination levels by administering vaccines at school-based venues in an atmosphere that students find to be safe and non-judgmental. Government programs need to extend their models to be able to serve boys and girls using well-organized parental consent programs and after-school programs.

Culturally Sensitive Education to Address Myths, Concerns, and Misinformation

A nationwide publicity effort targeting diverse audiences should begin. Educational programs must customize their messages to be sensitive to the cultural and religious norms of the location and emphasize cancer prevention more than sexual activity. The vaccine normalization process demands participation from media influencers alongside healthcare professionals, religious scholars, and educators to confront misinformation about the vaccine. Schools should enhance the quality and breadth of their sexual health education programs. Teaching comprehensive sexual health information alongside vaccination information to students through school curricula can help adolescents better grasp HPV and STI dangers and their protective benefits. A basic understanding of vaccine science enables people to make knowledgeable choices and decreases negative perceptions about immunization. Similarly, trusted figures like imams, community elders, and nurses need to be informed about the necessity of HPV vaccination to disseminate accurate information and address vaccine hesitancy within their communities.

Empowering Healthcare Workers as Advocates for Male HPV Vaccination

Empowering healthcare workers (HCWs) as advocates for male HPV vaccination is crucial for increasing vaccination rates and reducing HPV-related diseases in both males and females. HCWs are trusted sources of health information, and their recommendations significantly influence vaccine acceptance. By providing HCWs with the necessary knowledge, skills, and resources, they can effectively promote HPV vaccination among males, contributing to broader public health goals. Healthcare providers should receive specialized training to become advocates for vaccines. Ongoing professional training about male HPV risks and vaccination advantages must be provided to physicians, nurses, and pharmacists. The expansion of culturally sensitive communication tools among health providers creates opportunities for confident dialogue with parents who delay or resist vaccination decisions and their adolescent children.

Investing in Conducting Local Research and Surveillance

A common theme from the review is that data on HPV prevalence is not readily available or may be underreported. As such, all MENA countries need to support research investigating HPV prevalence throughout genders across their populations. Accurate prevalence statistics, together with risk factor assessments and vaccine effectiveness information, will help build sound health policies along with effective outreach messaging and support the case for complete population vaccination.

## Conclusions

In summary, HPV vaccination among males in the UAE and MENA region is an underutilized public health prevention tool in the fight against HPV related cancers, from cervical cancer to oropharyngeal and male genital cancer. Observing and reviewing the trend of these cancers acts as a tool to understand and address barriers to vaccinations, which is an imperative investment. HPV vaccine acceptance across the MENA region, especially in countries like Saudi Arabia, the UAE, and Qatar, faces several sociocultural and informational barriers. In order to address these obstacles, it is essential to roll out consistent regional strategies to achieve equitable vaccine coverage, develop distribution guidelines, provide financial resources, and introduce comprehensive monitoring mechanisms. The cooperation between the state apparatus, medical institutes, and global agencies may help to create a more overall vision of the implementation of HPV immunization programs. Further, religious issues, societal norms, and misconceptions about HPV and its vaccination require special consideration in every country in the GCC. Healthcare providers must receive training in good communication strategies to communicate on various issues and develop confidence with patients. A combination of different techniques and constant surveillance can assist in countering the worrisome effects of rising transmission of HPV and the rising cases of HPV-associated cancers.

## References

[1] Williamson AL (2023). Recent developments in human papillomavirus (HPV) vaccinology. Viruses.

[2] Patra S, Shand H, Ghosal S, Ghorai S (2025). HPV and male cancer: pathogenesis, prevention, and impact. J Oman Med Assoc.

[3] (2025). WHO updates recommendations on HPV vaccination schedule. http://www.who.int/news/item/20-12-2022-WHO-updates-recommendations-on-HPV-vaccination-schedule.

[4] (2025). Human papillomavirus (HPV) vaccines. https://www.cancer.gov/about-cancer/causes-prevention/risk/infectious-agents/hpv-vaccine-fact-sheet.

[5] Obeid D, Alsuwairi F, Alnemari R (2024). Sexually transmitted infections in the middle east and North Africa: comprehensive systematic review and meta-analysis. BMC Infect Dis.

[6] Alshahrani NZ, Alshahrani JA, Almushari BS (2024). Parental perspectives on human papillomavirus (HPV) vaccination in Gulf Cooperation Council Countries: a systematic review. Medicine (Baltimore).

[7] Farahat FM, Faqih NT, Alharbi RS, Mudarris RI, Alshaikh SA, Al-Jifree HM (2021). Epidemiological characteristics of cervical cancer in a tertiary care hospital, western Saudi Arabia: a retrospective record-based analysis from 2002-2018. Saudi Med J.

[8] Bruni L, Albero G, Rowley J (2023). Global and regional estimates of genital human papillomavirus prevalence among men: a systematic review and meta-analysis. Lancet Glob Health.

[9] AlMesbah N, Maatoug J, Selim N, Bougmiza I (2024). Human papillomavirus prevalence and genotypes in Gulf Cooperation Council countries: a scoping review 2017-2024. Qatar Med J.

[10] Chesson HW, Dunne EF, Hariri S, Markowitz LE (2014). The estimated lifetime probability of acquiring human papillomavirus in the United States. Sex Transm Dis.

[11] Rodríguez-Álvarez MI, Gómez-Urquiza JL, Husein-El Ahmed H, Albendín-García L, Gómez-Salgado J, Cañadas-De la Fuente GA (2018). Prevalence and risk factors of human papillomavirus in male patients: a systematic review and meta-analysis. Int J Environ Res Public Health.

[12] Kim S, Bae S (2024). The necessity of human papillomavirus vaccination in men: a narrative review. Urogenital Tract Infect.

[13] Gamaoun R (2018). Knowledge, awareness and acceptability of anti-HPV vaccine in the Arab states of the Middle East and North Africa Region: a systematic review. East Mediterr Health J.

[14] Farsi NJ, Baharoon AH, Jiffri AE, Marzouki HZ, Merdad MA, Merdad LA (2021). Human papillomavirus knowledge and vaccine acceptability among male medical students in Saudi Arabia. Hum Vaccin Immunother.

[15] (2025). Ministry of Health and Prevention adopts proactive HPV prevention strategy promoting UAE’s leadership in the Eastern Mediterranean. https://mohap.gov.ae/en/w/ministry-of-health-and-prevention-adopts-proactive-hpv-prevention-strategy-promoting-uae-s-leadership-in-the-eastern-mediterranean.

[16] Mahmoud I, Al Eid MM, Mohamed MA, Aladwani AJ, El Amin NE (2024). Human papillomavirus vaccination and Pap test uptake, awareness, and barriers among young adults in Gulf Cooperation Council countries: a comparative cross-sectional survey. J Infect Public Health.

[17] Darraj AI, Arishy AM, Alshamakhi AH (2022). Human papillomavirus knowledge and vaccine acceptability in Jazan Province, Saudi Arabia. Vaccines (Basel).

[18] Hendaus MA, Hassan M, Alsulaiti M (2021). Parents attitudes toward the human papilloma virus (HPV) vaccine: a new concept in the State of Qatar. J Family Med Prim Care.

[19] Husain Y, Alalwan A, Al-Musawi Z, Abdulla G, Hasan K, Jassim G (2019). Knowledge towards human papilloma virus (HPV) infection and attitude towards its vaccine in the Kingdom of Bahrain: cross-sectional study. BMJ Open.

[20] Trangerud HA (2023). "What is the problem with vaccines?" A typology of religious vaccine skepticism. Vaccine X.

[21] Zuzak TJ, Zuzak-Siegrist I, Rist L, Staubli G, Simoes-Wüst AP (2008). Attitudes towards vaccination: users of complementary and alternative medicine versus non-users. Swiss Med Wkly.

[22] Alhusayn KO, Alkhenizan A, Abdulkarim A, Sultana H, Alsulaiman T, Alendijani Y (2022). Attitude and hesitancy of human papillomavirus vaccine among Saudi parents. J Family Med Prim Care.

[23] Saqer A, Ghazal Sh, Barqawi H, Babi JA, AlKhafaji R, Elmekresh MM (2017). Knowledge and awareness about cervical cancer Vaccine (HPV) among parents in Sharjah. Asian Pac J Cancer Prev.

[24] Altom F, Khawaji NY, Almalki MM, Almohammadi WA, Al-Enezi HS, Al-Khalil SY (2024). Knowledge, attitude, and perception regarding the human papillomavirus (hpv) vaccine among parents at Al-Madinah Al-Munawwar: a cross-sectional study. Cureus.

[25] Salleh NS, Abdullah KL, Chow HY (2025). Cultural barriers and facilitators of the parents for human papillomavirus (HPV) vaccination uptake by their daughters: a systematic review. J Pediatr (Rio J).

[26] Tobaiqi MA, Albouq RA, Ban AM (2024). Knowledge, awareness, and attitudes towards hpv and its vaccination among women in the medina region: a cross-sectional study. Healthcare (Basel).

[27] Al Saedi RA, Al Daajani MM, Alghamdi SA (2024). Parental awareness, attitude, and acceptability of the human papillomavirus vaccine in Jeddah, Saudi Arabia: an analytical cross-sectional study. Cureus.

[28] Alsulami FT (2024). Exploring the impact of knowledge about the human papillomavirus and its vaccine on perceived benefits and barriers to human papillomavirus vaccination among adults in the western region of Saudi Arabia. Healthcare (Basel).

[29] Taumberger N, Joura EA, Arbyn M, Kyrgiou M, Sehouli J, Gultekin M (2022). Myths and fake messages about human papillomavirus (HPV) vaccination: answers from the ESGO Prevention Committee. Int J Gynecol Cancer.

[30] Alkhalili M, Al-Hmaid Y, Kheirallah K, Mehaisen L (2024). Assessment of knowledge of sexual reproductive health among female university students in Jordan. Cureus.

[31] AlMusawi S, Radhi M, Mani Prabu Kumar T (2025). Vaccine knowledge, attitude, and hesitancy among healthcare professionals in the Kingdom of Bahrain: a cross-sectional survey. Cureus.

[32] Vincent SC, Al Yaquobi S, Al Hashmi A (2024). A systematic review of knowledge, attitudes, and factors influencing HPV vaccine acceptance among adolescents, parents, teachers, and healthcare professionals in the Middle East and North Africa (MENA) region. Cureus.

[33] (2025). HPV vaccination in MENA. https://opalbiopharma.com/hpv-vaccination-in-mena/.

[34] Kisa S, Kisa A (2024). Religious beliefs and practices toward HPV vaccine acceptance in Islamic countries: a scoping review. PLoS One.

